# Knowledge, Awareness and Practices Related to Indoor Air Quality Among University Students in Ras Al Khaimah, United Arab Emirates: A Cross-Sectional Study

**DOI:** 10.3390/ijerph23040478

**Published:** 2026-04-09

**Authors:** Raqshan Wajih Siddiqui, Tabish Wajih Siddiqui, Fatema Marwan Alzaabi, Asma Abdullah Alzaabi, Manal Mahmoud Sami

**Affiliations:** 1 Rak College of Medical Sciences, Ras Al Khaimah Medical and Health Sciences University, Ras Al Khaimah P.O. Box 11172, United Arab Emirates; raqshanwajih@gmail.com (R.W.S.); asma.binhumaidan@gmail.com (A.A.A.); manal@rakmhsu.ac.ae (M.M.S.); 2 Emirates Health Services, Ras Al Khaimah P.O. Box 2299, United Arab Emirates; 3 Dubai Health, Mohammed Bin Rashid University, Dubai P.O. Box 505055, United Arab Emirates; 4 Seha Sheikh Khalifa Medical City, Abu Dhabi P.O. Box 51900, United Arab Emirates

**Keywords:** environmental pollutants, universities, cross-sectional studies, United Arab Emirates, health education, global health

## Abstract

**Highlights:**

**Public health relevance—How does this work relate to a public health issue?**

- Assesses indoor air quality (IAQ) knowledge, awareness, and practices among university students in the United Arab Emirates, a population with prolonged indoor exposure.
- Addresses gaps in environmental health literacy related to invisible pollutants and long-term health risks in rapidly urbanizing settings.

**Public health significance—Why is this work of significance to public health?**

- Identifies low objective IAQ knowledge and limited awareness of formal guidelines despite high perceived importance.
- Demonstrates a strong association between knowledge levels and engagement in protective behaviors.

**Public health implications—What are the key implications or messages for practitioners, policy makers and/or researchers in public health?**

- Supports integrating structured IAQ education into university curricula to improve environmental health literacy.
- Highlights the need for institutional and policy-level initiatives to promote evidence-based IAQ practices in academic settings.

**Abstract:**

Indoor air quality (IAQ) is a critical determinant of environmental health, yet awareness among young adults in rapidly urbanizing regions remains unclear. This study assessed knowledge, awareness, and practices related to IAQ among university students in Ras Al Khaimah, United Arab Emirates, and compared outcomes between medical and non-medical disciplines, while examining associations between knowledge levels and IAQ-related behaviors. A cross-sectional survey was conducted among 386 undergraduate students from three universities using a pre-validated, self-administered questionnaire. Overall, 52.1% of participants had heard of IAQ. Appropriate knowledge (≥60%) was demonstrated by 26.9% of students, and only 3.4% achieved high knowledge (≥80%). Medical students were significantly more likely than non-medical students to demonstrate appropriate knowledge (38.1% vs. 18.3%; *p* = 0.001), and female students scored higher than males (32.8% vs. 20.3%; *p* = 0.006). Awareness of IAQ guidelines was limited (65.3% unaware). Although 85.2% reported engaging in at least one IAQ-improving behavior, practices were mainly limited to ventilation and avoidance of indoor smoking. Higher knowledge levels were significantly associated with protective behaviors (*p* < 0.001). These findings indicate limited objective knowledge despite moderate recognition of IAQ importance, underscoring the need for structured educational interventions to enhance environmental health literacy.

## 1. Introduction

Indoor air quality (IAQ), defined by the physical, chemical, and biological characteristics of air within enclosed environments, profoundly influences human health, comfort, and productivity [1]. The World Health Organization (WHO) estimates that indoor air pollution is responsible for approximately 3.2 million deaths annually, predominantly from cardiovascular and respiratory diseases [2]. Although this burden is most pronounced in low- and middle-income countries due to household fuel combustion, IAQ remains a significant concern in urban areas of higher-income nations, where inadequate ventilation, volatile organic compounds (VOCs), and emissions from synthetic building materials contribute to indoor pollutant exposure [3]. While earlier studies primarily focused on identifying pollutant sources and measuring concentration levels, more recent research highlights the complex interplay between exposure patterns, building design, socioeconomic factors, and behavioral determinants [3]. Recent work has also shifted toward occupant-centered and behavior-informed IAQ management, showing that perceptions, response efficacy, and user engagement can strongly influence how indoor environments are experienced and managed [4]. However, methodological discrepancies and regional biases have limited the comparability of findings, leaving uncertainty regarding how awareness and behaviors influence IAQ-related health outcomes.

With individuals spending nearly 90% of their time indoors, IAQ is recognized as a critical determinant of both public health and health equity [5]. Despite this, public understanding remains limited and uneven worldwide. Studies across Latin America, Asia, and Africa consistently report low awareness of indoor pollutants, associated health risks, and preventive strategies [6]. However, these studies employ heterogeneous methodologies, ranging from qualitative assessments to structured surveys with varying knowledge indices, resulting in inconsistent estimates of awareness and limiting cross-regional comparability. Although they collectively highlight inadequate public knowledge, particularly among younger populations, differences in environmental context and policy frameworks complicate direct comparisons. This suggests a persistent gap in effective communication and education surrounding indoor environmental health.

This gap is particularly concerning among young adults, especially university students, whose future professional roles may influence public health practices, environmental policies, and societal norms. Despite relatively high levels of formal education, existing evidence suggests that IAQ awareness in this group is often fragmented and may not translate into accurate knowledge or evidence-based behaviors [7]. Some studies indicate that individuals in health-related disciplines demonstrate greater awareness; however, direct comparisons between medical and non-medical students using standardized measures remain limited, and the extent to which knowledge informs practical behavior is not well established [8]. Strengthening IAQ literacy in this population is therefore essential, both to improve immediate health behaviors and to foster long-term environmental health resilience.

In the Middle East, IAQ challenges are intensified by the convergence of climatic, cultural, and structural factors. The region’s arid climate, frequent dust storms, and heavy reliance on air conditioning systems contribute to indoor pollutant accumulation, while rapid urbanization and prolonged time spent in enclosed, mechanically ventilated environments further increase exposure risks [9,10]. Recent United Arab Emirates (UAE) studies have reported that indoor pollutant concentrations in homes can exceed recommended levels, with factors such as cooking, smoking, closed-window air-conditioning use, and limited ventilation contributing to elevated exposure [11]. Although some studies in the Gulf region have examined IAQ parameters such as particulate matter, ventilation performance, and thermal comfort, these investigations have largely focused on environmental monitoring and engineering aspects, with comparatively little attention to public awareness and behavioral dimensions [9]. Recent reviews from the Gulf region similarly emphasize health risks and building-related exposure factors, but they also highlight the limited number of studies addressing occupants’ knowledge, perceptions, and everyday practices [12]. Consequently, there remains a limited understanding of how individuals perceive and respond to IAQ risks in real-world settings. This gap is particularly evident in the UAE, where research on IAQ awareness and practices among university students, especially in rapidly developing emirates such as Ras Al Khaimah remains scarce [13].

Given this background, the present study aimed to evaluate IAQ knowledge, awareness, and practices among university students in Ras Al Khaimah, UAE. The specific objectives were as follows:

- Assess students’ knowledge of IAQ, including sources, health risks, and guideline awareness.
- Evaluate students’ awareness and recognition of common indoor pollutants and preventive measures.
- Examine self-reported IAQ-improving practices, such as ventilation, avoidance of indoor smoking, and use of air purifiers.
- Compare IAQ knowledge, awareness, and practices between medical and non-medical students to identify disciplinary differences and opportunities for targeted educational interventions.

This study makes a novel contribution by providing the first comprehensive assessment of IAQ literacy among university students in Ras Al Khaimah, highlighting disciplinary differences between medical and non-medical students. Methodologically, it employs a structured, bilingual questionnaire capturing both knowledge and practice behaviors, offering a replicable framework for similar populations. The findings advance understanding of IAQ awareness in a region with unique climatic and cultural conditions, and provide actionable insights for educational and public health interventions.

## 2. Materials and Methods

### 2.1. Study Design

This study employed a descriptive, cross-sectional design using a structured survey to assess knowledge, awareness, and practices regarding IAQ among undergraduate students. The cross-sectional approach allowed for the evaluation of these variables at a single point in time across a diverse student population, providing a snapshot of current awareness levels and behavioral practices. The survey-based methodology was chosen for its efficiency in reaching a large number of students across multiple institutions and for its suitability in capturing self-reported data on knowledge, perceptions, and behaviors related to IAQ.

### 2.2. Study Setting

The study was conducted at three universities located in Ras Al Khaimah, United Arab Emirates: Ras Al Khaimah Medical and Health Sciences University (RAKMHSU), the American University of Ras Al Khaimah (AURAK), and the University of Bolton. These institutions were selected to ensure representation from students enrolled in both medical and non-medical academic programs, thereby capturing a diverse range of educational backgrounds.

For the purposes of this study, students were categorized into medical and non-medical groups based on their field of study. Medical students were defined as those enrolled in healthcare-related programs, including medicine, dentistry, pharmacy, and allied health sciences such as nursing and other health-focused disciplines. Non-medical students were defined as those enrolled in programs not directly related to healthcare, including but not limited to engineering, business, humanities, and other non-health disciplines.

The inclusion of multiple universities facilitated comparison between these groups and enabled the assessment of potential differences in knowledge, awareness, and practices related to indoor air quality (IAQ). Collectively, these institutions host a heterogeneous student population in terms of age, gender, and cultural background, providing an appropriate setting for examining IAQ-related perceptions and behaviors in a real-world educational context.

### 2.3. Study Duration

Data collection was conducted over a period of six months, from December 2023 to May 2024. This timeframe allowed sufficient opportunity for participant recruitment across all universities, ensuring adequate sample size and diversity. The extended data collection period also enabled follow-up reminders to maximize response rates and provided flexibility in addressing logistical challenges, such as academic schedules, holidays, and examination periods. The duration was considered appropriate for capturing a representative cross-section of the student population while minimizing temporal bias in reporting IAQ knowledge and practices.

### 2.4. Study Population and Eligibility

All male and female undergraduate students enrolled at the participating universities were eligible for inclusion, regardless of ethnic background or year of study. Students from universities outside the selected institutions and those who did not complete the survey were excluded. Participation was voluntary, and informed consent was implied through completion and submission of the questionnaire.

The inclusion of participants aged below 18 years reflects the presence of early-entry undergraduate students within the participating institutions. These individuals were included to ensure comprehensive representation of the undergraduate population. Ethical considerations for this subgroup were addressed through the study’s approval by institutional ethics committees, which permitted inclusion of minors under minimal-risk survey conditions. Participation was voluntary and anonymous, and completion of the questionnaire was taken as implied consent. As no identifiable personal data were collected and the study posed no foreseeable risk, additional written parental or guardian consent was waived in accordance with institutional ethical guidelines.

### 2.5. Patient and Public Involvement

University students were involved in this study as participants through the completion of the survey. Their responses directly informed the analysis and interpretation of findings related to knowledge, awareness, and practices regarding indoor air quality.

### 2.6. Sample Size and Sampling Method

The required sample size was calculated using the standard formula:

$$n=\frac{Z^{2}\times P(1-P)}{d^{2}}$$

where *n* represents the estimated sample size, *P* is the assumed proportion of students with adequate IAQ knowledge, *Z* is the Z-score corresponding to a 95% confidence level (1.96), and *d* is the allowable margin of error (0.05). Based on this calculation, the minimum sample size was 384 participants. A total of 386 students participated in the study.

The survey employed a non-probability convenience sampling strategy, whereby participants were recruited through university-affiliated email lists, social media platforms, and peer networks across the three selected universities. Out of the total student population of 3000 (1200 students from RAKMHSU, 1200 students from AURAK, and 600 students from the University of Bolton), a total of 386 students responded, yielding an estimated response rate of approximately 12.9%. While efforts were made to ensure representation across institutions and academic disciplines, formal probability-based techniques such as stratified random sampling were not implemented. To partially mitigate sampling bias, the survey distribution targeted students proportionally across universities and fields of study.

### 2.7. Data Collection Instrument

Data were collected using a structured, self-administered questionnaire adapted from a previously validated instrument used in Singapore [14]. The questionnaire was available in English and Arabic and contained a total of 14 items. These included 4 questions related to demographic information, 3 questions assessing knowledge of IAQ, 5 questions assessing awareness of IAQ, and 2 questions assessing practices aimed at improving IAQ, such as ventilation, avoidance of indoor smoking, and use of air purifiers. The instrument covered IAQ knowledge, sources of awareness, recognition of indoor pollutants and associated health risks, guideline knowledge, and behavioral practices, providing a comprehensive assessment of students’ knowledge, awareness, and practices regarding IAQ. These were structured as multiple-response questions, where each correct option selected contributed to the total score, while incorrect or unselected correct options did not contribute. The cumulative knowledge score had a maximum of 47 points, reflecting the total number of correct response options across all knowledge items. The full questionnaire administered in this study is provided in Table A1 (Appendix A).

### 2.8. Validation

The questionnaire was pilot tested on 10 students to assess clarity and usability. Feedback from the pilot study informed minor revisions to wording and structure. The internal consistency of the questionnaire was evaluated using Cronbach’s alpha, which yielded a value of 0.83, indicating good reliability.

### 2.9. Study Variables

Independent variables included demographic characteristics (age, gender, and university type) and sources of IAQ awareness. Dependent variables comprised knowledge scores, self-reported IAQ-related health problems, and adoption of IAQ-improving practices.

A composite knowledge score (maximum of 47 points) was developed to assess recognition of indoor pollutants, understanding of associated health consequences, and awareness of IAQ guidelines. Scores were categorized based on Bloom’s modified classification, a widely used framework in knowledge–attitude–practice studies, and aligned with the rigorous standards set by Montuori et al. (2023): high (≥80%), appropriate (60–79%), and inappropriate (<60%) [15,16]. While Montuori et al. applied multiple linear regression to identify demographic predictors, the present analysis focused specifically on the relationship between academic background and the transition from knowledge to practice. Behavioral practices were evaluated based on engagement in IAQ-promoting actions. IAQ-improving behaviors were operationally defined as self-reported engagement (yes/no) in predefined actions listed in the questionnaire, including opening windows for ventilation, avoiding indoor smoking, and using air purifiers. These variables were assessed as binary responses and did not capture frequency, duration, or intensity of the behaviors.

### 2.10. Data Collection Procedure

Participants were recruited using convenience sampling, with the questionnaire distributed via university email, social media platforms, and peer networks to ensure proportional representation across universities and disciplines. Participants were provided with an information sheet detailing the study’s objectives and procedures. Informed consent was obtained electronically before participants could proceed to the questionnaire. Responses were automatically recorded in a secure database.

### 2.11. Statistical Analysis

Data was exported to Microsoft Excel (Microsoft Corporation, Redmond, WA, USA) and analyzed using Statistical Package for Social Sciences (SPSS) version 29 (IBM Corp., Armonk, NY, USA). Descriptive statistics, including means, standard deviations, frequencies, and percentages, were used to summarize participant characteristics and responses. Associations between knowledge scores, demographic variables, and IAQ practices were examined using chi-square tests for categorical variables and independent *t*-tests for continuous variables. To control for potential confounding, multivariable logistic regression analysis was performed to identify independent predictors of appropriate knowledge, adjusting for age, gender, and university type. Adjusted odds ratios (AORs) with 95% confidence intervals (CIs) and *p*-values were reported. Statistical significance was set at *p* < 0.05. Results were presented in tables to clearly demonstrate the relationships between demographics, knowledge, awareness, and IAQ-related behaviors. 

### 2.12. Ethical Considerations

This study adhered to the ethical standards set forth by the Helsinki Declaration of 1964 and its subsequent revisions, including the 2013 update. Ethical approval was obtained from the RAKMHSU’s Research & Ethics Committee (RAKCOMS-REC-27-2023/24-UG) and the Ministry of Health and Prevention Research Ethics Committee (MOHAP) (MOHAP/REC/2023/43-2023-UG-M). Informed consent was obtained from all participants prior to their inclusion in the study. Participants were assured of the confidentiality of their responses and their right to withdraw from the study at any time without consequences.

## 3. Results

### 3.1. Participant Demographics

A total of 386 participants completed the survey. Respondents were drawn from three universities: RAKMHSU (*n* = 168, 43.5%), AURAK (*n* = 168, 43.5%), and University of Bolton (*n* = 50, 13.0%). Medical students accounted for 43.5% (*n* = 168), and non-medical students for 56.5% (*n* = 218). The gender distribution was slightly skewed toward females (52.8%) compared to males (47.2%).

Participants’ ages ranged from 15 to 43 years, with a mean of 20.3 ± 2.5 years. The majority were young adults aged 18–24 years (92.0%, *n* = 355), while only 4.1% (*n* = 16) were younger than 18 years, and 3.9% (*n* = 15) were over 24 years (Table 1). These demographics provided a representative sample for examining IAQ awareness and practices across both medical and non-medical student populations.

### 3.2. Awareness and Perceived Importance of IAQ

Overall, 52.1% of respondents reported awareness of the concept of IAQ prior to the survey. Awareness was slightly higher among medical students (56.0%) than non-medical students (49.1%), although this difference was not statistically significant (*p* = 0.114). Mean awareness scores on a 10-point scale were 4.94 ± 2.68 for medical students and 4.55 ± 2.64 for non-medical students, indicating moderate awareness overall.

Gender differences were minimal, with females reporting slightly higher scores than males (4.88 ± 2.55 vs. 4.54 ± 2.77; *p* = 0.538). Respondents over 24 years had the highest awareness (5.87 ± 2.59), compared with younger groups (<18 years: 4.88 ± 3.34; 18–24 years: 4.66 ± 2.63), though differences were not significant (*p* = 0.706).

Participants rated IAQ importance highly, with a mean score of 7.74 ± 2.45 across all groups. Perceived importance did not differ significantly by gender, age, or university type (Table 2).

### 3.3. Sources of IAQ Awareness, Recognized Pollutants, and Perceived Health Risks

The primary sources of IAQ awareness were online platforms or websites (30.0%), followed by mass media such as television, newspapers, and radio (23.6%). Academic sources, including university coursework, accounted for 12.4%, while family or friends contributed 10.9%. Notably, 23.0% of respondents could not identify a source of their IAQ knowledge, suggesting gaps in structured IAQ education (Table 3).

Regarding pollutants, respondents identified secondhand smoke (18.3%), carbon monoxide (17.5%), and mold or other biological contaminants (16.1%) as the most common IAQ hazards. Dust (14.2%), particulate matter (PM) (10.6%), VOCs (7.5%), and radon (5.2%) were less frequently recognized. Additionally, 10.4% mentioned other unspecified pollutants, indicating variability in hazard perception.

In terms of health risks, respondents cited respiratory illness (15.9%), nasal symptoms (14.1%), eye irritation (12.9%), throat irritation (11.3%), headaches (11.8%), and dizziness (10.6%). Fatigue (8.2%), cancer (8.3%), and other unspecified effects (7.9%) were also reported, reflecting the broad health impacts attributed to poor IAQ (Table 3).

### 3.4. Self-Reported Health Problems and Awareness of Guidelines

Overall, 38.6% of participants reported experiencing health problems they suspected were related to poor IAQ. Medical students reported a slightly higher prevalence (41.1%) than non-medical students (36.7%), though this difference was not statistically significant (*p* = 0.381). Gender did not significantly influence self-reported IAQ-related health issues (female: 38.2%; male: 39.0%; *p* = 0.876). By age group, respondents over 24 years reported the highest prevalence (66.7%), compared to 37.7% in the 18–24-year group and 31.3% in those under 18 years (Table 4). Although a higher prevalence of IAQ-related health problems was observed among participants aged >24 years, this did not reach statistical significance (χ2 *p* = 0.065). All expected cell counts were adequate; however, Fisher’s exact test was additionally performed due to the small subgroup size and yielded consistent non-significant results (*p* = 0.072).

Awareness of IAQ guidelines from health organizations was limited, with 65.3% of respondents reporting no awareness. Medical students were slightly more aware than non-medical students (38.7% vs. 31.7%; *p* = 0.150), while age and gender showed no significant differences.

Most respondents (83.4%) reported that poor IAQ negatively affects academic performance and concentration, with similar perceptions across all groups (Table 4).

### 3.5. Knowledge Levels and IAQ-Improving Practices

Knowledge regarding IAQ was generally low. Only 26.9% of respondents demonstrated appropriate knowledge (≥60% on the knowledge score), while 73.1% had inappropriate knowledge (<60%).

We supplemented this categorical data with detailed score distributions. The mean knowledge score among all students was 23.3 ± 8.3 (range: 3–43). By subgroup, medical students had significantly higher mean knowledge scores compared with non-medical students (25.0 ± 8.7 vs. 22.0 ± 7.7; mean difference = 3.1, *p* < 0.001). Females scored higher than males (24.36 ± 8.49 vs. 22.08 ± 7.87), and scores by age group were 20.81 ± 11.02 for ≤17 years, 23.30 ± 8.18 for 18–24 years, and 25.53 ± 6.96 for ≥25 years.

Medical students were also significantly more likely to have appropriate knowledge than non-medical students (38.1% vs. 18.3%; *p* = 0.001). Similarly, female respondents were more knowledgeable than males (32.8% vs. 20.3%; *p* = 0.006), while age did not significantly affect knowledge scores (*p* = 0.396) (Table 5).

After adjusting for potential confounders (age and gender), medical students remained significantly more likely to have appropriate knowledge compared with non-medical students (AOR = 2.49; 95% CI: 1.54–4.01; *p* < 0.001). Female students showed a borderline significant, higher likelihood of appropriate knowledge compared with male students (AOR = 1.63; 95% CI: 0.999–2.65; *p* = 0.050), while age group was not significantly associated with knowledge (Table 6).

Knowledge level was strongly associated with engagement in positive IAQ practices. Among participants with appropriate knowledge, 96.2% reported performing such practices, compared to 80.1% with inappropriate knowledge (*p* < 0.001). Reported measures included opening windows or ventilating rooms (39.0%), avoiding indoor smoking (27.2%), and using air purifiers (25.0%), while less common actions included using indoor plants and limiting chemical cleaners. Only 8.8% reported engaging in no IAQ-improving practices (Table 7).

## 4. Discussion

This study assessed university students’ knowledge, awareness, and practices regarding IAQ in Ras Al Khaimah, UAE. Although most recognized IAQ’s importance for health and academic performance, few had adequate knowledge, and nearly two thirds were unaware of formal guidelines, indicating that awareness is largely conceptual and insufficient to support evidence-based practices. This distinction is critical, as it suggests that the recognition of IAQ does not equate to the ability to apply appropriate preventive measures. As a result, IAQ-related behaviors may be inconsistent and guided by incomplete or non-scientific understanding rather than evidence-based decision-making.

### 4.1. Comparison with Previous Research

The results of this study are consistent with previous regional and international findings. Al-Khamees (2018) reported similarly low levels of IAQ awareness among students and staff at Kuwait University, highlighting the persistence of this issue across academic institutions in the Gulf [17]. Ravi et al. (2025) likewise observed limited understanding and suboptimal attitudes toward indoor pollution among university populations, indicating that environmental health literacy remains underdeveloped even among educated groups [18]. However, the present study extends these findings by demonstrating that even when awareness is present, it does not necessarily translate into adequate or actionable knowledge. This suggests that prior studies may have overestimated functional understanding by relying on awareness as a proxy for knowledge.

At a global level, Adamopoulos et al. (2025) identified poor IAQ in educational settings as a neglected public health concern associated with respiratory and cognitive effects [19]. Studies from Singapore and Hungary have further shown that higher educational exposure is associated with improved environmental literacy, supporting the finding that medical students in this study demonstrated greater awareness and more responsible practices than their non-medical counterparts [14,20]. Nevertheless, the observation that a substantial proportion of medical students still lacked adequate knowledge highlights potential gaps in current health-related curricula, where environmental determinants of health may not be sufficiently emphasized in a practical or applied context.

Environmental investigations corroborate students’ perceptions. Assessments of IAQ in university classrooms have reported elevated concentrations of carbon dioxide, PM, and formaldehyde, particularly in poorly ventilated spaces [21,22]. Fuentes-Ferragud et al. (2024) additionally identified volatile and semi-volatile organic compounds with potential toxicological implications [23]. Together, this body of evidence underscores that IAQ challenges in academic settings are both perceptible and objectively measurable. This alignment suggests that while students may recognize poor air quality, they may lack the necessary knowledge to accurately identify pollutant sources or implement effective mitigation strategies. In line with this, Kureshi et al. (2023) demonstrated that improved awareness and environmental monitoring can positively influence IAQ-related behaviors, reinforcing the association between knowledge and preventive practices observed in this study [24].

### 4.2. Determinants of Knowledge and Practice

Medical students and female participants demonstrated higher knowledge scores, consistent with prior evidence suggesting that health-related curricula and gender-linked health literacy influence awareness and preventive behaviors [24,25,26]. The higher knowledge levels among female students may be explained by health education exposure and gender-specific traits that often foster greater engagement in health-promoting behaviors, cleanliness, and adherence to preventive practices. This is supported by previous studies indicating that female students are more likely to prioritize health-related topics and adopt relevant behaviors [27,28,29,30]. Despite this advantage, more than 60 percent of medical students failed to achieve adequate knowledge levels, indicating that IAQ remains underrepresented even within health sciences education. Gender differences similarly reflect broader systemic gaps, as a substantial proportion of female students also demonstrated insufficient understanding [31]. These findings suggest that while certain subgroups demonstrate relatively higher awareness, IAQ literacy remains inadequate at a population level, indicating a systemic gap rather than isolated disparities.

Reported sources of IAQ knowledge reflect a pronounced digital shift. Online platforms were the dominant source of information, while traditional media played a secondary role. Formal academic coursework was reported by only a small minority of participants, and nearly one quarter identified no source of IAQ knowledge [17]. This reliance on digital platforms presents both opportunities and risks, as information accessibility is increased but may vary in accuracy and credibility, potentially contributing to inconsistent understanding. This highlights limited institutional integration of environmental health education and emphasizes the potential of digital channels for targeted awareness initiatives.

### 4.3. Knowledge Practice Gap

Although most students reported engaging in behaviors to improve IAQ, these practices were largely confined to basic measures such as opening windows, avoiding indoor smoking, and using air purifiers. These behaviors are consistent with previous studies reporting the most commonly adopted IAQ practices among students and the general population, and are also recommended by the WHO as effective strategies for reducing indoor air pollutants [2,32]. A significant positive correlation between knowledge and protective behaviors was observed, indicating that education enhances both the likelihood and quality of preventive actions. Students with higher knowledge were more likely to adopt evidence-informed practices, whereas those with limited knowledge relied on superficial measures. 

The gap between knowledge and comprehensive, guideline-based action reflects the complexity of behavior adoption in health contexts. Protective practices are influenced not only by knowledge but also by motivation, perceived susceptibility, and environmental constraints, such as building design and limited access to monitoring tools [32,33].

### 4.4. Health Implications

Limited awareness of less visible pollutants such as PM, VOCs, and radon is concerning given their established long-term health effects [34,35,36]. While acute symptoms were commonly recognized, chronic outcomes including asthma, cardiovascular disease, and malignancy were poorly understood, likely reflecting reliance on non-academic information sources [37]. The underrecognition of these less perceptible risks may lead to underestimation of long-term exposure and delayed adoption of preventive measures.

In university environments where students spend prolonged periods indoors, inadequate IAQ may negatively affect respiratory health, cognitive performance, and overall wellbeing [38,39,40]. This positions IAQ as not only a public health concern but also an academic and institutional issue, with potential implications for student productivity and educational outcomes.

### 4.5. Global Relevance

The findings of this study have both regional and global relevance. Indoor air pollution is a major public health concern worldwide, affecting billions and contributing to respiratory and cardiovascular disease, cognitive impairment, and long-term morbidity [41,42]. Although research has largely focused on occupational and residential settings, university populations represent a critical yet underexplored group due to prolonged time spent in classrooms and dormitories, often with inadequate ventilation. The gaps identified in Ras Al Khaimah may reflect similar trends in other low- and middle-income countries, as well as among young adults in high-income settings where awareness of indoor environmental risks remains limited.

The patterns observed in this study suggest that gaps in IAQ awareness among university students may not be unique to Ras Al Khaimah but may reflect broader global trends, particularly in settings where environmental health education is not systematically integrated into curricula.

Universities play a key role in shaping future professionals and decision-makers. Integrating IAQ literacy into curricula can influence behaviors beyond campus, extending to households and workplaces. The growing reliance on digital platforms for health information highlights opportunities for online and social media-based interventions to improve IAQ awareness globally [43,44]. Such approaches may generate broader improvements in environmental health literacy across populations [45].

### 4.6. Implications for Policy and Education

The findings highlight the need for universities and policymakers to improve IAQ literacy. Integrating IAQ education into both medical and non-medical curricula can address knowledge gaps, equipping students with theoretical understanding and practical skills. Curriculum content should cover pollutant identification, health impacts, guideline-based interventions, and hands-on learning, including air quality monitoring.

From a theoretical perspective, the findings demonstrate that awareness alone is not an adequate indicator of environmental health literacy and should not be used as a sole measure in future research. From a practical standpoint, the strong association between knowledge and behavior supports the implementation of structured educational interventions to improve IAQ-related practices. At the policy level, incorporating IAQ standards and educational requirements into higher education frameworks may help institutionalize environmental health literacy.

Interactive strategies such as gamified learning, virtual simulations, and workshops can enhance engagement and retention. Embedding IAQ education contributes to Sustainable Development Goals 3, 4, and 11 [46,47,48].

Campus-wide initiatives leveraging digital platforms and social media can complement curricular efforts by providing updates on IAQ, preventive strategies, and the impact of individual actions. Universities may also consider infrastructural improvements, including well-ventilated classrooms, monitoring technologies, and enhanced dormitory air management, which support health outcomes and experiential learning. Policy-level incorporation of IAQ standards into national higher education frameworks, aligned with UAE Vision 2031, can institutionalize environmental literacy [49,50]. Evidence from this study demonstrates a strong link between knowledge and protective behaviors, highlighting the value of structured education [51]. Combining education, awareness campaigns, and structural interventions can foster environmentally literate graduates who practice and promote preventive measures, ultimately improving indoor air quality and overall wellbeing.

### 4.7. Limitations

This cross-sectional study limits causal inference between knowledge and behavior. Self-reported data may introduce recall or social desirability bias. The sample was restricted to three universities in Ras Al Khaimah, which limits generalizability; therefore, caution should be exercised when extrapolating these findings to other regions of the UAE. Future multi-center, nationwide studies are recommended to provide a more representative assessment of IAQ knowledge and behaviors. The survey did not assess advanced technical understanding of IAQ guidelines, and age-related analyses were limited by small subgroup sizes. Additionally, certain potentially relevant variables, such as year of study and type of residence (e.g., on-campus dormitory versus off-campus housing), were not captured and may influence students’ knowledge, awareness, and practices related to indoor air quality. For instance, senior students may have greater exposure to health-related coursework, while residential environment may affect both exposure to indoor pollutants and IAQ-related behaviors. IAQ-improving behaviors were assessed as self-reported binary measures without quantification of frequency or duration, which may introduce subjective interpretation and limit the assessment of behavior intensity.

## 5. Conclusions

This study provides novel insights into the relationship between the knowledge of IAQ and the adoption of protective behaviors among university students. While higher knowledge was associated with a greater likelihood of engaging in preventive actions, the findings reveal that awareness alone does not consistently translate into comprehensive behavioral change. This underscores the importance of considering additional factors, including individual motivation, ingrained habits, and environmental constraints, which can either facilitate or hinder the practical application of knowledge. By identifying these gaps, the study highlights the limitations of educational interventions that focus solely on information dissemination and emphasizes the need for multifaceted strategies that combine education with behavioral support and environmental modifications. Furthermore, these results contribute to public health practice by providing evidence-based guidance for designing targeted programs that can more effectively promote sustained protective behaviors, ultimately reducing exposure risks and enhancing indoor environmental quality. Beyond its immediate context, the study advances understanding of the complex interplay between knowledge, behavior, and contextual factors, offering a foundation for future research aimed at bridging the gap between awareness and action in IAQ and broader health promotion initiatives.

## Figures and Tables

**Table 1:** Demographic characteristics of respondents.

Characteristic	*n*	%	Mean ± SD (Where Applicable)
**University**			
RAKMHSU (Medical)	168	43.5	–
AURAK (Non-medical)	168	43.5	–
University of Bolton (Non-medical)	50	13.0	–
**University category**			
Medical	168	43.5	–
Non-medical	218	56.5	–
**Gender**			
Male	182	47.2	–
Female	204	52.8	–
**Age group**			20.3 ± 2.5 years (range 15–43)
<18 years	16	4.1	–
18–24 years	355	92.0	–
>24 years	15	3.9	–

**Table 2:** Awareness, perceived importance, and mean scores on IAQ.

Variable	*n*	% Aware	Mean Awareness (1–10) ± SD	*p*-Value	Mean Importance (1–10) ± SD	*p*-Value
**Overall**	386	52.1	4.75 ± 2.66	–	7.74 ± 2.45	–
**University category**						
Medical	168	56.0	4.94 ± 2.68	0.114	7.78 ± 2.49	0.908
Non-medical	218	49.1	4.55 ± 2.64		7.71 ± 2.46	
**Gender**						
Male	182	48.3	4.54 ± 2.77	0.538	7.42 ± 2.51	0.199
Female	204	55.3	4.88 ± 2.55		8.03 ± 2.41	
**Age group**						
<18 years	16	50.0	4.88 ± 3.34	0.706	6.56 ± 3.14	0.131
18–24 years	355	52.1	4.66 ± 2.63		7.80 ± 2.42	
>24 years	15	53.3	5.87 ± 2.59		7.67 ± 2.82	

**Table 3:** Sources of awareness, pollutants recognized, and health risks associated with poor IAQ.

Sources of Awareness	*n* (%)	Pollutants Recognized	*n* (%)	Reported Health Risks	*n* (%)
Online platforms/websites	116 (30.0)	Secondhand smoke	71 (18.3)	Respiratory illness	61 (15.9)
Mass media (TV, radio, press)	91 (23.6)	Carbon monoxide	68 (17.5)	Nasal symptoms	54 (14.1)
Academic sources	48 (12.4)	Mold/biological contaminants	62 (16.1)	Eye irritation	49 (12.9)
Family/friends	42 (10.9)	Dust	55 (14.2)	Throat irritation	43 (11.3)
No identifiable source	89 (23.0)	PM	41 (10.6)	Headaches	45 (11.8)
–	–	VOCs	29 (7.5)	Dizziness	41 (10.6)
–	–	Radon	20 (5.2)	Fatigue	31 (8.2)
–	–	Other (unspecified)	40 (10.4)	Cancer	32 (8.3)
–	–	–	–	Other	30 (7.9)

**Table 4:** Self-reported health problems, awareness of IAQ guidelines, and perceived academic impact by demographic group.

Variable	Category (*n* = Number of Students)	IAQ-Related Health Problems Yes *n* (%)	*p*-Value	Aware of IAQ Guidelines Yes *n* (%)	*p*-Value	Perceive Academic Impact Yes *n* (%)	*p*-Value
**Overall**	201	149 (38.6%)	-	134 (34.7%)	-	322 (83.4%)	-
**Age group**	<18 years (16)	5 (31.3)	0.065	6 (37.5)	0.588	13 (81.3)	0.541
	18–24 (355)	134 (37.7)		121 (34.1)		298 (83.9)	
	>24 (15)	10 (66.7)		7 (46.7)		11 (73.3)	
**Gender**	Male (182)	71 (39.0)	0.876	66 (36.3)	0.546	148 (81.3)	0.294
	Female (204)	78 (38.2)		68 (33.3)		174 (85.3)	
**University type**	Medical (168)	69 (41.1)	0.381	65 (38.7)	0.15	144 (85.7)	0.287
	Non-medical (218)	80 (36.7)		69 (31.7)		178 (81.7)	

**Table 5:** Distribution of knowledge levels by demographic characteristics.

Variable	Category (*n*)	Appropriate Knowledge *n* (%)	Inappropriate Knowledge *n* (%)	Mean ± SD (Range)	*p*-Value
Overall	–	104 (26.9)	282 (73.1)	23.3 ± 8.3 (3–43)	–
University type	Medical (168)	64 (38.1)	104 (61.9)	25.0 ± 8.7 (3–43)	0.001
	Non-medical (218)	40 (18.3)	178 (81.7)	22.0 ± 7.7 (3–43)	–
Gender	Female (204)	67 (32.8)	137 (67.2)	24.36 ± 8.49 (3–43)	0.006
	Male (182)	37 (20.3)	145 (79.7)	22.08 ± 7.87 (4–43)	–
Age group	≤17 (16)	3 (18.8)	13 (81.3)	20.81 ± 11.02 (3–37)	0.396
	18–24 (355)	95 (26.8)	260 (73.2)	23.30 ± 8.18 (3–43)	–
	≥25 (15)	6 (40.0)	9 (60.0)	25.53 ± 6.96 (12–35)	–

**Table 6:** Adjusted analysis of factors associated with knowledge.

Variable	AOR (95% CI)	Adjusted *p*-Value
Medical vs. Non-medical	2.49 (1.54–4.01)	<0.001
Female vs. Male	1.63 (0.999–2.65)	0.050
Age 18–24 vs. <18	0.27 (0.05–1.44)	0.125
Age ≥25 vs. <18	0.46 (0.15–1.39)	0.168

**Table 7:** Association between knowledge levels and IAQ-improving practices.

Knowledge Level	Positive Practices *n* (%)	Negative Practices *n* (%)	Total	*p*-Value
Appropriate Knowledge (≥60%)	100 (96.2)	4 (3.8)	104	<0.001
Inappropriate Knowledge (<60%)	226 (80.1)	56 (19.9)	282	–
Total	326	60	386	–

## Data Availability

The data supporting the findings of this study are available from the corresponding author upon reasonable request.

## Abbreviations

The following abbreviations are used in this manuscript:

IAQ	Indoor Air Quality
UAE	United Arab Emirates
RAKMHSU	Ras Al Khaimah Medical and Health Sciences University
AURAK	American University of Ras Al Khaimah
SPSS	Statistical Package for Social Sciences
WHO	World Health Organization
MOHAP	Ministry of Health and Prevention
REC	Research Ethics Committee
RAKCOMS-REC	Ras Al Khaimah College of Medical Sciences Research Ethics Committee
PM	Particulate Matter
VOCs	Volatile Organic Compounds
SD	Standard Deviation
TV	Television
CO_2_	Carbon Dioxide
SDGs	Sustainable Development Goals

## Appendix A. Questionnaire

**Table A1:** Survey questionnaire used in the study.

Section	Question	Response Options
**Demographics**	Age	<18/18–24/>24
**Demographics**	Gender	Male/Female
**Demographics**	University	Medical (RAKMHSU)/Non-medical (AURAK & University of Bolton)
**Demographics**	Currently living in Ras Al Khaimah	Yes/No
**Knowledge**	Have you heard of the term “Indoor Air Quality” (IAQ) before taking this survey?	Yes/No
**Knowledge**	Which of the following air pollutants are you aware of that can affect IAQ?	Secondhand smoke, Carbon monoxide, Mold/biological contaminants, Dust, Particulate matter (PM), Volatile organic compounds (VOCs), Radon, Other (unspecified), I am not aware
**Knowledge**	What are the potential health risks associated with poor IAQ that you are aware of?	Respiratory illness, Nasal symptoms, Eye irritation, Throat irritation, Headaches, Dizziness, Fatigue, Cancer, Other
**Awareness**	On a scale of 1–10, how aware are you of the concept of IAQ?	1–10
**Awareness**	What sources have contributed to your awareness of IAQ?	Online platforms/websites, Mass media (TV, radio, press). Academic sources, Family/friends, No identifiable source
**Awareness**	On a scale of 1–10, how important do you believe IAQ is for your health and well-being?	1–10
**Awareness**	Are you aware of any specific IAQ guidelines or recommendations provided by health authorities?	Yes/No
**Awareness**	Do you think poor IAQ can impact academic performance and concentration in indoor study environments?	Yes/No
**Practices**	Have you personally experienced any health problems that you suspect may be related to indoor air quality?	Yes/No
**Practices**	Have you taken any actions to improve IAQ in your living or study spaces?	Opening windows; Using air purifiers; Avoiding smoking indoors; No action

## References

[1] US EPA (2024). Introduction to Indoor Air Quality. https://www.epa.gov/indoor-air-quality-iaq/introduction-indoor-air-quality.

[2] World Health Organization (2018). Household Air Pollution and Health. https://www.who.int/en/news-room/fact-sheets/detail/household-air-pollution-and-health.

[3] Chowdhury S., Pillarisetti A., Oberholzer A., Jetter J., Mitchell J., Cappuccilli E., Aamaas B., Aunan K., Pozzer A., Alexander D. (2023). A global review of the state of the evidence of household air pollution’s contribution to ambient fine particulate matter and their related health impacts. Environ. Int..

[4] Veiga I., Oliveira T., Naranjo-Zolotov M., Martins R., Karatzas S. (2025). Indoor air quality perception: Enablers and inhibitors of perceived occupant comfort. Environ. Int..

[5] Seguel J.M., Merrill R., Seguel D., Campagna A.C. (2016). Indoor Air Quality. Am. J. Lifestyle Med..

[6] Vardoulakis S., Giagloglou E., Steinle S., Davis A., Sleeuwenhoek A., Galea K.S., Dixon K., Crawford J.O. (2020). Indoor exposure to selected air pollutants in the home environment: A systematic review. Int. J. Environ. Res. Public Health.

[7] Rendon-Marin S., Higuita-Gutiérrez L.F., Gomez-Gallego D.M. (2024). Knowledge, Attitudes, and Practices Regarding Air Pollution among Medical Students. Int. J. Environ. Res. Public Health.

[8] Alves R.F. (2024). The relationship between health-related knowledge and attitudes and health risk behaviours among Portuguese university students. Glob. Health Promot..

[9] Amoatey P., Omidvarborna H., Baawain M.S., Al-Mamun A. (2018). Indoor air pollution and exposure assessment of the gulf cooperation council countries: A critical review. Environ. Int..

[10] Abdou Y., Ki Kim Y., Bande L. (2020). Indoor environmental quality evaluation in a hot and arid climate: A case study of a higher education office building. E3S Web Conf..

[11] Bani Mfarrej M.F., Qafisheh N.A., Bahloul M.M. (2020). Investigation of Indoor Air Quality inside Houses From UAE. Air Soil Water Res..

[12] Abusin S., Al-Thani N., Al-Emadi N., Petcu C. (2022). Health impact of indoor air pollution in the gulf region: A review. World J. Adv. Res. Rev..

[13] Yeatts K.B., El-Sadig M., Leith D., Kalsbeek W., Al-Maskari F., Couper D., Funk W.E., Zoubeidi T., Chan R.L., Trent C.B. (2012). Indoor Air Pollutants and Health in the United Arab Emirates. Environ. Health Perspect..

[14] Unni B., Tang N., Cheng Y.M., Gan D., Aik J. (2022). Community knowledge, attitude and behaviour towards indoor air quality: A national cross-sectional study in Singapore. Environ. Sci. Policy.

[15] Shi Z., Chen Y.B. (2025). Knowledge, Attitude, and Practice Toward Chronic Pain in Older Adults Among Health Sciences Students: A Cross-Sectional Study. J. Pain Res..

[16] Montuori P., Gioia M., Sorrentino M., Di Duca F., Pennino F., Messineo G., Maccauro M.L., Riello S., Trama U., Triassi M. (2023). Determinants Analysis Regarding Household Chemical Indoor Pollution. Toxics.

[17] Al-Khamees N.A. (2018). Knowledge of, attitudes toward, and practices regarding indoor pollution at Kuwait University. J. Geosci. Environ. Prot..

[18] Ravi R.K., Edwin V., Muthu P., Priya S.P. (2025). Awareness and perceptions about indoor air pollution among health professional students: Evidence from a cross-sectional survey from the Northern Emirates of the United Arab Emirates. Open Environ. Res. J..

[19] Adamopoulos I.P., Syrou N.F., Mijwil M., Thapa P., Ali G., Dávid L.D. (2025). Quality of indoor air in educational institutions and adverse public health in Europe: A scoping review. Electron. J. Gen. Med..

[20] Zsóka Á., Szerényi Z.M., Széchy A., Kocsis T. (2013). Greening due to environmental education? Environmental knowledge, attitudes, consumer behavior and everyday pro-environmental activities of Hungarian high school and university students. J. Clean. Prod..

[21] Elattar S.M., Abdullah I.D., Gebriel A.A.S. (2025). Evaluation of the impact of air pollutants on university indoor air quality: A case study. Front. Built Environ..

[22] Caciora T., Ilieş A., Berdenov Z., Al-Hyari H.S., Ilieş D.C., Safarov B., Hassan T.H., Herman G.V., Hodor N., Bilalov B. (2024). Comprehensive analysis of classroom microclimate in context to health-related national and international indoor air quality standards. Front. Public Health.

[23] Fuentes-Ferragud E., López A., Piera J.M., Yusà V., Garrigues S., de la Guardia M., Labrador F.X.L., Camaró M., Ibáñez M., Coscollà C. (2024). Indoor air quality and bioaerosols in Spanish university classrooms. Toxics.

[24] Kureshi R.R., Thakker D., Mishra B.K., Barnes J. (2023). From raising awareness to a behavioural change: A case study of indoor air quality improvement using IoT and COM-B Model. Sensors.

[25] Rababah J.A., Al-Hammouri M.M., Drew B.L., Aldalaykeh M. (2019). Health literacy: Exploring disparities among college students. BMC Public Health.

[26] Shah L.C., West P., Bremmeyr K., Savoy-Moore R.T. (2010). Health literacy instrument in family medicine: The “Newest Vital Sign” ease of use and correlates. J. Am. Board Fam. Med..

[27] Kabir A., Roy S., Begum K., Kabir A.H., Miah M.S. (2021). Factors influencing sanitation and hygiene practices among students in a public university in Bangladesh. PLoS ONE.

[28] Kligler B., Pinto Zipp G., Rocchetti C., Secic M., Ihde E.S. (2021). The impact of integrating environmental health into medical school curricula: A survey-based study. BMC Med. Educ..

[29] Vasileva P., Golubev V.Y., Ibragimov I., Rubtsova S. (2021). Trash to treasure: Integrating environmental awareness into university curriculum. J. Teach. Engl. Specif. Acad. Purp..

[30] Baba A., Shahrour I., Baba M. (2024). Indoor Environmental Quality for Comfort Learning Environments: Case Study of Palestinian School Buildings. Buildings.

[31] Tong V., Raynor D.K., Aslani P. (2025). Gender Differences in Health and Medicine Information Seeking Behaviour—A review. Umedumt.

[32] Frick M., Neu L., Liebhaber N., Sperner-Unterweger B., Stötter J., Keller L., Hüfner K. (2021). Why do we harm the environment or our personal health despite better knowledge? The knowledge action gap in healthy and climate-friendly behavior. Sustainability.

[33] Eriksson M., Sundberg L.R., Santosa A., Lindgren H., Ng N., Lindvall K. (2025). Health behavioural change—The influence of social-ecological factors and health identity. Int. J. Qual. Stud. Health Well-Being.

[34] US EPA (2021). Air Pollution and Cardiovascular Disease Basics. https://www.epa.gov/air-research/air-pollution-and-cardiovascular-disease-basics.

[35] US EPA (2019). Health Risk of Radon. https://www.epa.gov/radon/health-risk-radon.

[36] Nag P.K. (2018). Sick Building Syndrome and Other Building-Related Illnesses. Office Buildings: Health, Safety and Environment.

[37] Ramírez A.S., Ramondt S., Van Bogart K., Perez-Zuniga R. (2019). Public awareness of air pollution and health threats: Challenges and opportunities for communication strategies to improve environmental health literacy. J. Health Commun..

[38] Ezeamii V.C., Egbuchiem A.N., Obianyo C.M., Nwoke P., Okwuonu L. (2025). Air Quality Monitoring in Schools: Evaluating the Effects of Ventilation Improvements on Cognitive Performance and Childhood Asthma. Cureus.

[39] Harvard Healthy Buildings (2021). Impacts of Indoor Air Quality on Cognitive Function. https://healthybuildings.hsph.harvard.edu/impacts-of-indoor-air-quality-on-cognitive-function/.

[40] Tsaloglidou A., Koukourikos K., Pantelidou P., Kourkouta L. (2015). Indoor Air Quality and Health: Impact on Respiratory and Cardiovascular System. Int. J. Eng. Appl. Sci. (IJEAS).

[41] Tran V.V., Park D., Lee Y.C. (2020). Indoor Air Pollution, Related Human Diseases, and Recent Trends in the Control and Improvement of Indoor Air Quality. Int. J. Environ. Res. Public Health.

[42] Jakubowska A. (2023). The Burden of Air Pollution: A Perspective on Global Health Inequalities. Pollutants.

[43] Fallahi M.S., Faridzadeh A., Salahi M., Mehrabani R., Karimi H., Faraji A., Imanparvar S., Falahatian M., Bayat M., Norouzkhani N. (2024). Digital Health/e-Health Literacy among University Students in the COVID-19 Era: A Systematic Review. J. Med. Educ. Curric. Dev..

[44] Lim M.S.C., Molenaar A., Brennan L., Reid M., McCaffrey T. (2022). Young adults’ use of different social media platforms for health information: Insights from web-based conversations. J. Med. Internet Res..

[45] Muscat D., Morony S., Nutbeam D., Ayre J., Shepherd H., Smith S., Dhillon H., Hayen A., Luxford K., Meshreky W. (2020). Learners’ experience and perceived impact of a health literacy program in adult basic education: A qualitative study. Public Health Res. Pract..

[46] United Nations (2015). The 17 Sustainable Development Goals. https://sdgs.un.org/goals.

[47] US EPA (2014). Reference Guide for Indoor Air Quality in Schools. https://www.epa.gov/iaq-schools/reference-guide-indoor-air-quality-schools.

[48] Gomes M.I., Barreiros A.M., Pinto I., Rodrigues A. (2025). Improving Indoor Air Quality in a Higher-Education Institution Through Biophilic Solutions. Sustainability.

[49] The National Air Quality Agenda 2031—The Official Portal of the UAE Government. https://u.ae/en/about-the-uae/strategies-initiatives-and-awards/strategies-plans-and-visions/environment-and-energy/the-national-air-quality-agenda-2031.

[50] Smith C., Drinkwater A., Modlich M., Van der Horst D., Doherty R. (2024). IAQ and environmental health literacy: Lived experiences of vulnerable people. Build. Cities.

[51] Lindsey M., Chen S.R., Ben R., Manoogian M., Spradlin J. (2021). Defining Environmental Health Literacy. Int. J. Environ. Res. Public Health.

